# Pediatric Adrenocortical Carcinoma Presenting With Virilization: A Case of a Low-Grade Tumor in a Young Child

**DOI:** 10.7759/cureus.95946

**Published:** 2025-11-02

**Authors:** Ayed Askar, Shrouk F Mohamed, Mohammed Hussein Al-Mutair

**Affiliations:** 1 Department of Surgery, Idlib University Hospital, Idlib, SYR; 2 Department of Medicine, Alexandria Faculty of Medicine, Alexandria, EGY; 3 Department of Pediatric Endocrinology, Idlib University Hospital, Idlib, SYR

**Keywords:** children, precocious puberty (pp), s: adrenocortical carcinoma (acc), virilization, cushing’s syndrome

## Abstract

Adrenocortical carcinoma (ACC) is an uncommon and severe malignancy, particularly in the pediatric population. It often manifests with warning signs of hormonal imbalance, such as hirsutism and precocious puberty. Timely diagnosis is crucial for effective management.

We present the case of a 4-year-old female who presented with indications of virilization, including excessive hirsutism, acne, clitoromegaly, and a moon-shaped facial appearance, which raised suspicion of Cushing’s disease. An abdominal ultrasound and computed tomography angiography (CTA) were performed, identifying a left suprarenal tumor. Hormonal tests revealed elevated morning cortisol levels and reduced adrenocorticotropic hormone (ACTH) levels. The patient subsequently underwent total adrenalectomy.

Histopathological analysis confirmed a diagnosis of low-grade ACC with clear surgical margins. Pediatric ACC requires early detection to prevent invasion into adjacent structures. Careful observation of clinical symptoms, imaging findings, and histopathological features is crucial for accurate identification and appropriate therapy.

This report describes a rare case of pediatric ACC presenting with hormonal symptoms. Continued follow-up with laboratory testing, imaging, and symptom monitoring is essential to detect disease progression or recurrence, particularly in younger patients.

## Introduction

Adrenocortical carcinoma (ACC) is a rare and highly aggressive endocrine malignancy that develops in the cortex of the adrenal glands, which is responsible for producing steroid hormones. In the pediatric age group, ACC accounts for approximately 0.2% of all infant cancers, with an estimated incidence of 0.3-0.4 cases per million children per year. It is observed more frequently in girls than in boys and tends to occur between the ages of 0 and 10 years. ACC can be either benign or malignant and has significant associations with several hereditary cancer syndromes, such as Li-Fraumeni syndrome and Beckwith-Wiedemann syndrome [1,2].

Unlike adult cases, ACCs are more commonly identified in newborns and cause elevated levels of various hormones, primarily androgens and glucocorticoids. Children often present early with warning clinical signs resulting from abnormal hormonal fluctuations. They may present with excessive body hair, acne, clitoromegaly, excessive weight gain, moon face, and hypertension [2,3].

Diagnosis usually involves a combination of hormonal testing, imaging, and tissue biopsy. Imaging typically reveals a large mass above the kidney, often with areas of tissue necrosis or hemorrhage; however, in many cases, there is no local invasion or metastasis at the time of diagnosis [4,5].

Surgical resection remains the cornerstone of treatment; complete excision with total adrenalectomy offers the best chance for cure. Adjuvant therapy with chemotherapy or radiation may be considered in cases of incomplete resection or metastasis. Close follow-up is essential to monitor for recurrence or the development of new hormonal imbalances. Histological findings, such as tumor invasion into surrounding tissue or blood vessels, a high number of mitotic figures, and cellular atypia, help differentiate malignant ACC from benign adenomas [4,6].

This case adheres to the Surgical Case Report (SCARE) guidelines [7] and illustrates the clinical urgency and diagnostic complexity of this rare childhood malignancy.

## Case presentation

A 4-year-old female presented with poor growth, obesity, abnormal hair growth, and acne. On physical examination, the child’s general condition was excellent, and her blood pressure and heart rate were within normal limits. The parents reported notable weight gain, with her body weight above the 95th percentile. She had a moon face, one of the characteristic features of Cushing’s syndrome, and signs of virilization in the form of pustular acne, hirsutism, axillary and pubic hair, and clitoromegaly. However, there was no evidence of abdominal distension. The family reported no history of malignancy.

The patient’s hormonal profile revealed normal thyroid-stimulating hormone (TSH) and follicle-stimulating hormone (FSH) levels, with a mildly reduced luteinizing hormone (LH). Morning cortisol was elevated, while adrenocorticotropic hormone (ACTH) remained within the normal range. This pattern indicated autonomous cortisol secretion by the adrenal gland, as elevated cortisol levels would typically suppress ACTH through negative feedback if the source were pituitary or ectopic. The marked elevation of dehydroepiandrosterone sulfate (DHEA-SO₄) further supported an adrenal origin, as this hormone is primarily produced in the adrenal cortex. Insulin-like growth factor 1 (IGF-1) was reduced, consistent with the hormonal imbalance, as summarized in Table 1.

**Table 1:** Hormonal profile of the patient.

Hormone	Patient Value	Reference Range	Interpretation
Thyroid-stimulating hormone (TSH)	3.25 μIU/mL	0.4-4.0 μIU/mL	Normal
Follicle-stimulating hormone (FSH)	2.44 μIU/mL	1.5-12 μIU/mL	Normal
Luteinizing hormone (LH)	0.108 μIU/mL	1.0-9.0 μIU/mL	Slightly low
Morning cortisol	31.91 μg/dL	5-25 μg/dL	Elevated
Adrenocorticotropic hormone (ACTH)	41.5 pg/mL	10-60 pg/mL	Normal
Dehydroepiandrosterone sulfate (DHEA-SO₄)	>750 μg/dL	35-430 μg/dL	Elevated
Insulin-like growth factor 1 (IGF-1)	101.5 ng/mL	150-350 ng/mL	Low

**Figure 1:**
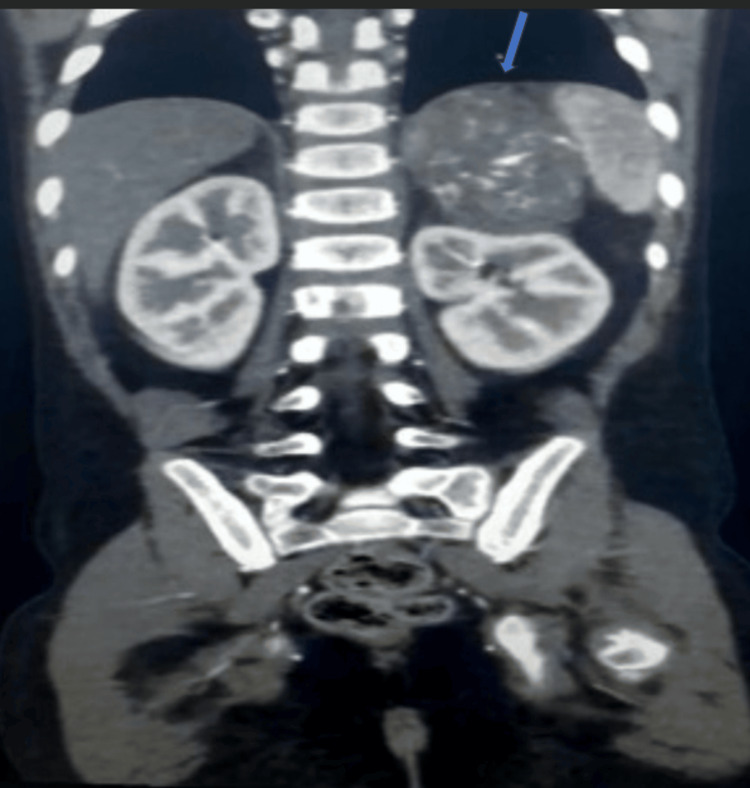
Computed tomography angiography (CTA) scan of the abdomen showing a large, heterogeneously enhancing mass (arrow) arising from the upper pole of the left kidney, consistent with an adrenal origin.

An abdominal ultrasound was performed, showing a solid tumor in the left suprarenal area. Computed tomography angiography (CTA) was subsequently performed to further evaluate the finding, confirming a substantial tumor in the left adrenal gland measuring 6 × 5 cm. It revealed a mass in the lateral suprarenal region accompanied by irregular low-density patches suggestive of tumor necrosis and central lysis (Figure 1). No invasion into adjacent organs or notable lymphadenopathy was detected. The right adrenal gland appeared normal, with no evidence of ascites or abnormal fluid accumulation.

**Figure 2:**
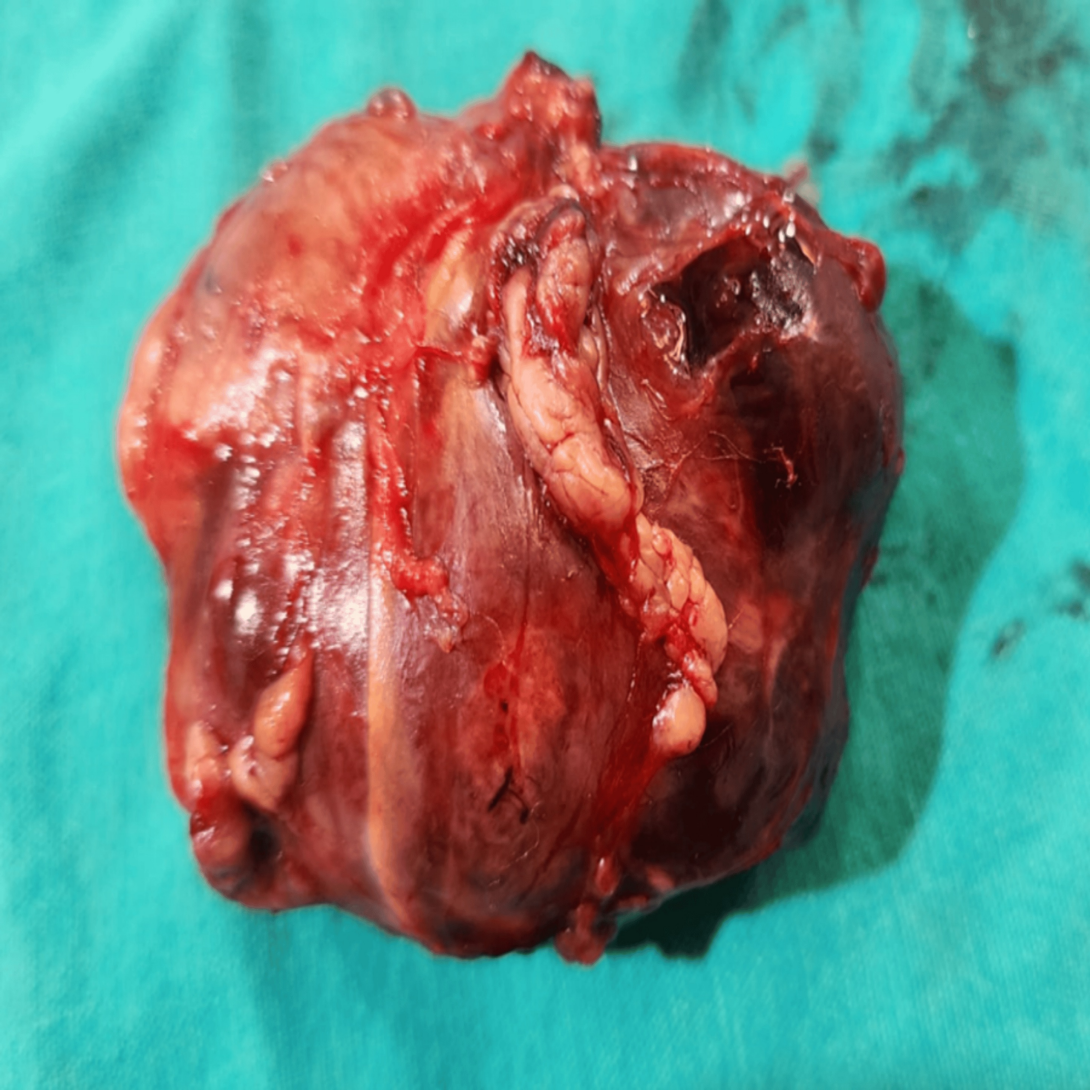
Macroscopic view of the excised adrenal tumor demonstrating a rounded, multilobulated soft-tissue mass with a smooth, glistening capsule and prominent surface vascular markings. The specimen shows patchy, dark hemorrhagic areas and a localized capsular defect, findings typical of malignant adrenal tumors.

The child’s overall condition was stable for surgery, and an adrenalectomy was performed via an open transabdominal approach through a left flank incision to provide optimal access to the inferior vena cava. The removed adrenal mass was a sizable adrenocortical tumor measuring approximately 6 × 5 × 4 cm. The mass was firm, grayish-brown in color, with rough outer surfaces (Figure 2). The patient remained in the pediatric intensive care unit until recovery, receiving hydrocortisone replacement therapy.

Histopathological examination of the cut sections revealed heterogeneous areas with evidence of necrosis and hemorrhage. Microscopically, the tumor consisted of an encapsulated mass featuring nests of varying sizes, large sheets, and trabeculae, demonstrating invasion of the dense fibrous capsule and lymphovascular invasion affecting both venous and sinusoidal structures. The tumor showed focal necrosis, hemorrhage, and degeneration, characterized by large pleomorphic cells with granular, transparent to eosinophilic cytoplasm, numerous intranuclear inclusions, and frequent mitoses, including atypical forms, with a mitotic rate of ≤20 mitoses per 50 high-power fields. No mesenchymal differentiation was detected, and discontinuity in the reticular fibers and basement membrane network was observed. The final diagnosis was consistent with low-grade ACC, with negative surgical margins following total adrenalectomy.

During postoperative follow-up, the child remained hospitalized for observation and was discharged once her laboratory results and vital signs returned to normal. One month later, she appeared clinically well, with a noticeable reduction in virilization.

## Discussion

For the pediatric population, ACC is an extremely rare and aggressive cancer that occurs more frequently among children under four years of age. ACC can be either a functioning or a non-functioning tumor, with nearly 95% of pediatric ACCs being functioning [8].

Numerous genetic factors may contribute to the risk of ACC, including abnormalities in β-catenin, insulin-like growth factor (IGF)-II/IGF-IR, and p53/Rb signaling pathways. Additionally, p53 mutations are frequent in these cases; therefore, genetic testing can be useful in investigating this tumor [4].

Most ACC cases present with virilization as the primary complaint. The most common signs include hirsutism, early growth spurts, acne, voice deepening, and the appearance of secondary sexual characteristics such as pubic hair, genital darkening, and enlargement of the penile or clitoral corpus cavernosum [2,4,5].

Our case demonstrated the typical signs of functional adrenocortical tumors, including features of Cushing’s syndrome, increased male characteristics, and a large adrenal mass confirmed by imaging and laboratory investigations. Comprehensive hormonal assessments are required in such cases, particularly for 17-hydroxyprogesterone (17-OH), dehydroepiandrosterone sulfate (DHEA), androstenedione, testosterone, 11-deoxycortisol, and cortisol, as these aid in diagnosing and classifying ACCs. Most cases show normal cortisol levels, whereas DHEA, testosterone, and 17-OH progesterone levels are typically elevated [9].

A CT scan is the most common imaging modality used to detect adrenal malignancies and to determine the stage and location of the tumor, as well as possible metastases [3,9]. Metastasis in adrenal tumors shows considerable variability, with reported rates ranging from 5% to 81%, most frequently affecting the liver, lungs, regional lymph nodes, kidneys, and bones, with lower likelihood in other sites. The recurrence rate in adults ranges from 60% to 70%; however, its prevalence in pediatric cases remains uncertain. Upon recurrence, surgical intervention should be considered, followed by pharmacological therapy to improve prognosis [4].

Several histopathological scoring systems, such as Weiss, Wieneke, and Van Slooten, are used to differentiate malignant from benign types of ACC [10]. Our case satisfied several Weiss criteria for malignancy histologically, including capsular and vascular invasion, necrosis, pleomorphic cells, and a high mitotic index with atypical mitoses. These findings supported the diagnosis of low-grade ACC based on clinical and imaging features. While no single histologic feature confirms malignancy, the constellation of findings in this case, particularly lymphovascular invasion and mitotic activity ≥20 per 50 high-power fields, is highly suggestive of aggressive biological behavior. In addition, the absence of distant metastasis or local invasion represents a favorable prognostic indicator.

The choice of a transabdominal adrenalectomy is recommended in pediatric patients, particularly when the tumor size exceeds 5 cm or when cortical carcinoma is suspected, to reduce the risk of intraoperative rupture and local recurrence [6].

Similar cases have been reported in the literature. Breim F et al. (2024) described a comparable case of ACC characterized by excessive hirsutism and clitoromegaly; the patient later died due to lung metastases, which were discovered late because of inadequate follow-up [11].

Another pediatric cohort study included five males and two females with ACC. All demonstrated excessive hormonal secretion, virilization, abnormal pubic hair growth, and genital enlargement. More than half (57%) exhibited Cushingoid features and hypertension. Biochemical analyses showed significantly elevated levels of DHEAS and testosterone, with decreased LH and FSH levels. Six of the seven participants demonstrated advanced bone age. Imaging revealed suprarenal masses in all patients, right-sided tumors in males and left-sided tumors in females, with no metastases at presentation. All patients underwent surgical excision. Wieneke scores ranged from 2 to 5, with four individuals having scores ≥4, indicative of malignancy. Despite elevated scores, only two children developed aggressive disease with recurrence and metastasis over an average follow-up of 12.8 months. Initially, no adjuvant therapy was administered; however, mitotane treatment was later introduced for the two metastatic cases. This highlights the need for rigorous postoperative monitoring every 6-8 weeks due to the unpredictable nature of juvenile ACC and the limited predictive ability of current histological scoring systems [12].

Al-Ghotani B et al. (2023) also reported a case of a 10-month-old girl presenting with a large right adrenal mass and Cushingoid symptoms. She died after two episodes of cardiac arrest following surgery [9].

Due to the tumor’s potential for recurrence and metastasis, consistent and thorough follow-up is essential to detect any signs of disease progression or relapse [9,11,12].

## Conclusions

This study presented an extremely rare case of ACC in a 4-year-old girl. Following left adrenalectomy, the patient demonstrated marked clinical improvement, with an immediate reduction in serum cortisol levels. This case underscores the critical importance of early diagnosis in preventing serious complications. ACC should be considered in the differential diagnosis of young children presenting with similar clinical features, warranting prompt and comprehensive evaluation.

## References

[1] Rodriguez-Galindo C, Figueiredo BC, Zambetti GP, Ribeiro RC (2005). Biology, clinical characteristics, and management of adrenocortical tumors in children. Pediatr Blood Cancer.

[2] Miele E, Di Giannatale A, Crocoli A (2020). Clinical, genetic, and prognostic features of adrenocortical tumors in children: a 10-year single-center experience. Front Oncol.

[3] Zagojska E, Malka M, Gorecka A, Ben-Skowronek I (2023). Case report: adrenocortical carcinoma in children-symptoms, diagnosis, and treatment. Front Endocrinol (Lausanne).

[4] Brondani VB, Fragoso MC (2020). Pediatric adrenocortical tumor - review and management update. Curr Opin Endocrinol Diabetes Obes.

[5] Gupta N, Rivera M, Novotny P, Rodriguez V, Bancos I, Lteif A (2018). Adrenocortical carcinoma in children: a clinicopathological analysis of 41 patients at the Mayo Clinic from 1950 to 2017. Horm Res Paediatr.

[6] Mihai R (2019). Open adrenalectomy. Gland Surg.

[7] Sohrabi C, Mathew G, Maria N, Kerwan A, Franchi T, Agha RA (2023). The SCARE 2023 guideline: updating consensus Surgical CAse REport (SCARE) guidelines. Int J Surg.

[8] Dasiewicz P, Moszczyńska E, Grajkowska W (2024). Adrenal cortical carcinoma: paediatric aspects - literature review. Pediatr Endocrinol Diabetes Metab.

[9] Al-Ghotani B, Alabdallah E, Shaaban V (2023). Adrenocortical carcinoma in a 10-month-old infant: a literature review and a rare case report. Ann Med Surg (Lond).

[10] Kerkhofs TM, Ettaieb MH, Verhoeven RH (2014). Adrenocortical carcinoma in children: first population-based clinicopathological study with long-term follow-up. Oncol Rep.

[11] Breim F, Jaweesh A, Sleibi A, Kanaa L, Sattout H, Morjan M (2024). Adrenocortical tumor manifesting as virilizing in a female child: Adenoma or carcinoma? A rare case report. Int J Surg Case Rep.

[12] Naotunna NP, Siriwardana HV, Lakmini BC (2023). Adrenocortical tumors in children: Sri Lankan experience from a single center, and a mini review. J Med Case Rep.

